# Residual Immunity from Smallpox Vaccination and Possible Protection from Mpox, China

**DOI:** 10.3201/eid3002.230542

**Published:** 2024-02

**Authors:** Yu Huang, Li Guo, Yanan Li, Lili Ren, Jiqin Nie, Fengwen Xu, Tingxuan Huang, Jingchuan Zhong, Zhangling Fan, Yin Zhang, Yu Xie, Qiao Zhang, Shan Mei, Yan Xiao, Xinming Wang, Liuhui Xu, Fei Guo, Jianwei Wang

**Affiliations:** NHC Key Laboratory of Systems Biology of Pathogens, National Institute of Pathogen Biology, Chinese Academy of Medical Sciences & Peking Union Medical College, Beijing, China (Y. Huang, L. Guo, Y. Li, L. Ren, J. Nie, F. Xu, T. Huang, J. Zhong, Z. Fan, Y. Zhang, Y. Xie, Q. Zhang, S. Mei, Y. Xiao, X. Wang, L. Xu, F. Guo, J. Wang);; Key Laboratory of Respiratory Disease Pathogenomics, Chinese Academy of Medical Sciences, Beijing (L. Guo, L. Ren, J. Wang);; National Institute of Pathogen Biology and Center for AIDS Research, Chinese Academy of Medical Sciences and Peking Union Medical College, Beijing (Y. Huang, F. Xu, Z. Fan, Y. Xie, S. Mei, F. Guo);; Christophe Mérieux Laboratory, National Institute of Pathogen Biology, Chinese Academy of Medical Sciences and Peking Union Medical College, Beijing (L. Guo, Y. Li, L. Ren, J. Nie, T. Huang, J. Zhong, Y. Zhang, Q. Zhang, Y. Xiao, X. Wang, L. Xu, J. Wang);; Key Laboratory of Pathogen Infection Prevention and Control (Ministry of Education), State Key Laboratory of Respiratory Health and Multimorbidity, National Institute of Pathogen Biology, Chinese Academy of Medical Sciences and Peking Union Medical College, Beijing (L. Guo, L. Ren, F. Guo);; National Key Laboratory of Immunity and Inflammation, Beijing (J. Wang)

**Keywords:** vaccinia virus, smallpox, mpox, monkeypox, viruses, neutralizing antibodies, memory B cell, memory T cell, vaccine-preventable diseases, China

## Abstract

Among persons born in China before 1980 and tested for vaccinia virus Tiantan strain (VVT), 28.7% (137/478) had neutralizing antibodies, 71.4% (25/35) had memory B-cell responses, and 65.7% (23/35) had memory T-cell responses to VVT. Because of cross-immunity between the viruses, these findings can help guide mpox vaccination strategies in China.

On July 23, 2022, the World Health Organization declared the global mpox outbreak to be a public health emergency of international concern (https://www.who.int/europe/news/item/23-07-2022-who-director-general-declares-the-ongoing-monkeypox-outbreak-a-public-health-event-of-international-concern). No specific treatment is currently approved for mpox. Vaccines such as JYNNEOS (Bavarian Nordic, https://www.bavarian-nordic.com) and ACAM2000 (Emergent BioSolutions Inc., https://www.emergentbiosolutions.com) are available for preexposure protection from mpox (*1*), and tecovirimat can be used for patients who are at risk for severe disease (*2*). 

Vaccinia virus Tiantan strain (VTT) was historically used for vaccines in the smallpox virus eradication campaign in China. Given the high level of sequence homology among their surface proteins, smallpox vaccination provided ≈85% protection against mpox (*3*). Because the World Health Organization declared that smallpox had been eradicated and routine use of vaccinia vaccine was terminated in most countries by 1980–1981, most persons born after 1980 do not have vaccinia virus–elicited immunity. Vaccinia-derived protection wanes in the vaccinated population over time, which may lead to an increase in susceptibility to monkeypox virus (MPXV) infection because of cross-immunity between the 2 viruses. 

After the first mpox case imported from Europe to mainland China on September 14, 2022 (*4*), investigation of the level of residual VTT-specific immunity in the population of China became pressing, as researchers assessed susceptibility to mpox and guided development of appropriate protective strategies. Different patterns of residual immunity against vaccinia suggest different strategies in responding to mpox transmission. However, levels of residual immunity to poxviruses in the population in China are not well assessed. We measured VTT-specific humoral and cellular immune responses in a diverse population born during 1930–2008 in China.

## The Study

In this cross-sectional cohort study, we collected blood specimens from 1,070 healthy donors who lived in Beijing, Shanxi Province, Heilongjiang Province, Hubei Province, or Shenzhen during regular health check-ups. Among the participants (Table 1), 478 were born during 1930–1979 and 592 were born during 1980–2008; ages ranged from 1 month to 90 years. The study was approved by the institutional review boards of the Chinese Academy of Medical Sciences’ Institute of Pathogen Biology (approval no. 2013-IPB-03, IPB-2021-15).

**Table 1:** Characteristics of 1,070 participants in a cross-sectional cohort study to determine IgG titers against vaccinia virus Tiantan strain, China*

Characteristic	Decade of birth, no. (%)							
	1930–1939	1940–1949	1950–1959	1960–1969	1970–1979	1980–1989	1990–2008	Total
Overall	106 (9.91)	104 (9.72)	76 (7.10)	95 (8.88)	97 (9.07)	84 (7.85)	508 (47.48)	1,070 (100)
Sex								
M	63 (5.89)	61 (5.70)	39 (3.64)	53 (4.95)	40 (3.74)	42 (3.93)	285 (26.64)	583 (54.49)
F	43 (4.02)	43 (4.02)	37 (3.46)	42 (3.93)	57 (5.33)	42 (3.93)	223 (20.84)	487 (45.51)

*Participants lived in Beijing, Shanxi Province, Heilongjiang Province, Hubei Province, or Shenzhen..

We tested serum samples from all participants to determine IgG titers against VTT by using ELISA. We performed a Gaussia luciferase-based vaccinia neutralization assay to determine the presence of neutralizing antibodies (NAbs). We performed memory B-cell and memory T-cell enzyme-linked immunospot (ELISpot) assays (Charles River Laboratories, https://www.criver.com) by using cryopreserved peripheral blood mononuclear cells (PBMCs). Because of insufficient PBMC samples, we evaluated memory B- and T-cell responses in a subgroup of the enrolled participants (Appendix Figure 1).

**Figure 1:**
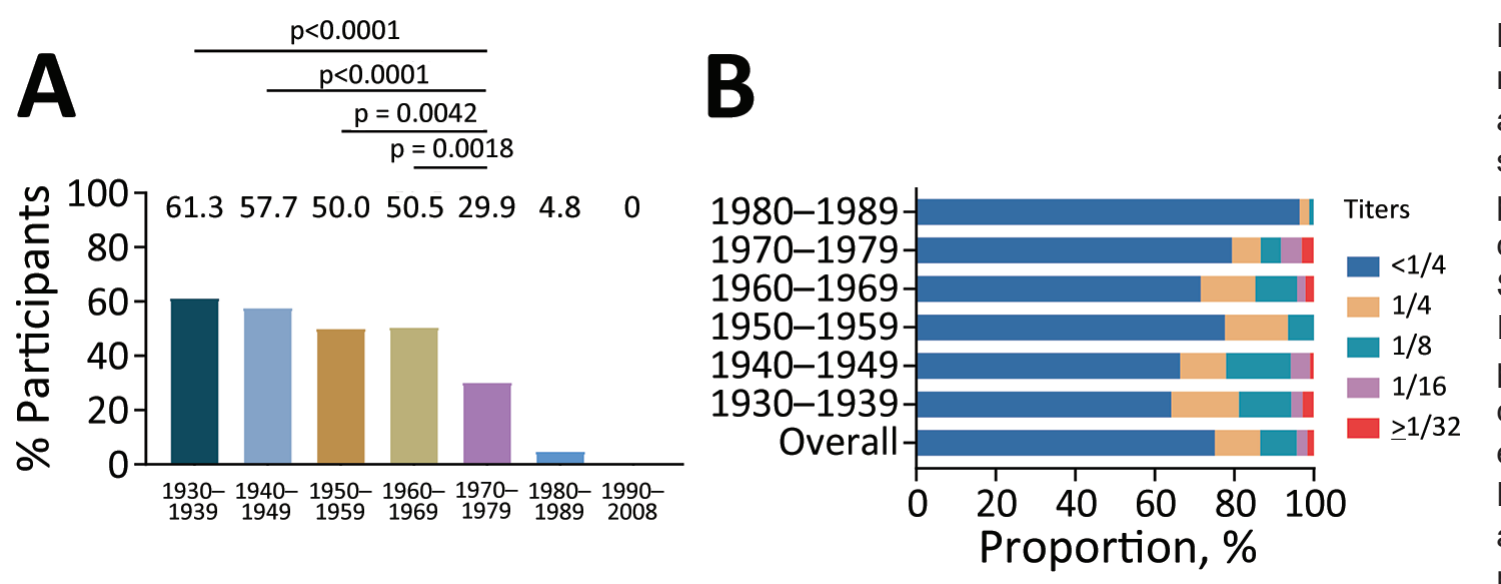
Serum IgG and neutralizing antibody responses against vaccinia virus Tiantan strain (VTT) among 1,070 participants in a cross-sectional cohort study, China. A) Seropositivity of VTT-specific IgG by birth cohort in 1,070 persons born during 1930–2008, conducted with χ^2^ or Fisher exact test as appropriate. B) Prevalence of neutralizing antibody by birth cohort in 562 persons born before 1990. .

Overall VTT seropositivity was 50.2% (240/478) in participants born before 1980. Persons born during 1970–1979 had the lowest seropositivity, 29.9% (29/97), compared with 61.3% (65/106 [p<0.0001]) among persons born during 1930–1939, 57.7% (60/104 [p<0.0001]) among persons born during 1940–1949, 50.0% (38/76 [p = 0.0042]) among persons born during 1950–1959, 50.5% (48/95 [p = 0.0018]) among persons born during 1960–1969. By comparison, ≈4.8% (4/84) participants born during 1980–1989 had VTT-specific IgG, and VTT-specific IgG was not detectable in persons born after 1990 (Figure 1, panel A). The VTT-specific IgG titers were not significantly different among participants born during 1930–1939, 1940–1949, 1950–1959, and 1960–1969 (p = 0.11), but all were higher than in persons born during 1970–1979 (Appendix Figure 2, panel A).

We examined distribution of NAb levels in relation to year of birth (Table 2; Figure 1, panel B). Of the 478 serum samples from persons born before 1980, most (341 [71.3%]) had an NAb titer of <1/4. Of the remaining samples, NAb titers were 1/4 for 62 (13.0%), 1/8 for 51 (10.7%), 1/16 for 15 (3.1%), and 1/32 for 9 (1.9%), suggesting the lack or low titers of NAb against VTT. VTT NAbs were detectable in 35.8% (38/106) of persons born during 1930–1939, 33.7% (35/104) born during 1940 –1949, 22.4% (17/76) born during 1950–1959, 28.4% (27/95) born during 1960–1969, and 20.6% (20/97) born during 1970–1979 but were detectable in only 3.6% (3/84) born during 1980–1989 (>1/4) (Appendix Figure 2, panel B). We observed a significant correlation between NAb and IgG titers in persons born before 1990 (Spearman r = 0.54; p<0.0001) (Appendix Figure 2, panel C).

**Table 2:** Neutralizing antibody titers against vaccinia virus Tiantan strain in persons born during 1930–1979, by birth cohort, China*

Decade of birth	Neutralizing antibody titers, no. (%)				
	<1/4	1/4	1/8	1/16	>1/32
1930–1939, n = 106	68 (64.2)	18 (17.0)	14 (13.2)	3 (2.8)	3 (2.8)
1940–1949, n = 104	69 (66.3)	12(11.5)	17 (16.3)	5 (4.8)	1 (0.96)
1950–1959, n = 76	59 (77.6)	12 (15.8)	5 (6.6)	0 (0)	0 (0)
1960–1969, n = 95	68 (71.6)	13 (13.7)	10 (10.5)	2 (2.1)	2 (2.1)
1970–1979, n = 97	77 (79.4)	7 (7.2)	5 (5.2)	5 (5.2)	3 (3.1)
Overall, N = 478	341 (71.3)	62 (13.0)	51 (10.7)	15 (3.1)	9 (1.9)

*Participants lived in Beijing, Shanxi Province, Heilongjiang Province, Hubei Province, or Shenzhen.

**Figure 2:**
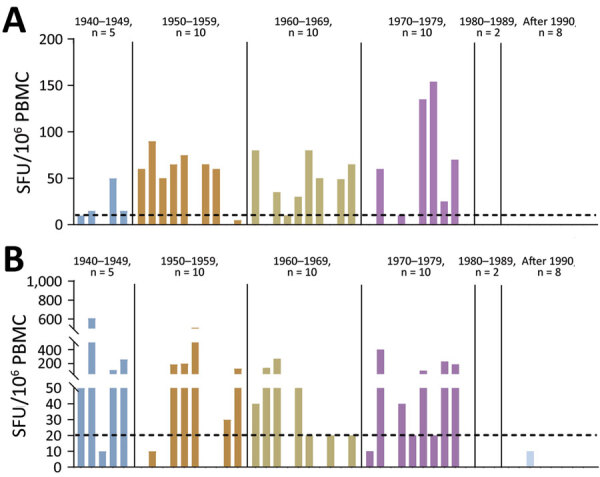
Vaccinia virus–specific memory B- and T-cell responses among 45 participants in a cross-sectional cohort study to determine IgG titers against vaccinia virus Tiantan strain (VTT), China. A) Magnitude of memory B-cell responses against VTT for each person. B) Magnitude of interferon-γ T-cell responses against VTT for each person. Dotted lines indicate detection limit of assay. PBMC, peripheral blood mononuclear cells; SFU, spot-forming units.

We measured VTT-specific memory B-cell responses in 45 participants whose PBMCs were isolated successfully (Appendix Figure 3). Approximately 71.4% (25/35) of persons born before 1980 showed VTT-specific memory B-cell responses; positivity across the 4 birth decades was 80% (4/5) for 1940–1949, 70% (7/10) for 1950–1959, 80% (8/10) for 1960–1969, and 60% (6/10) for 1970–1979. PBMCs of all persons born after 1980 were negative for VTT-specific memory B-cells (Figure 2, panel A). We observed no significant correlations between VTT-specific memory B-cell magnitude and VTT IgG (Appendix Figure 4, panel A) or NAb (Appendix Figure 4, panel B) titers.

We further evaluated interferon-γ (IFN-γ) responses to VTT in the same 45 participants (Appendix Figure 5). We detected VTT-specific memory T-cell responses in 65.7% (23/35) of persons across the 4 birth decades, distributed as 80% (4/5) for 1940–1949, 50% (5/10) for 1950–1959, 70% (7/10) for 1960–1969, and 70% (7/10) for 1970–1979. In contrast, T-cell IFN-γ responses were below the detection limit in the 10 persons born after 1980 (Figure 2, panel B). We observed no correlations between the magnitude of VTT-specific memory T-cell responses and IgG (Appendix Figure 6, panel A) or NAb (Appendix Figure 6, panel B) titers. 

As a control, we tested for influenza virus and Epstein Barr virus–specific memory T-cell responses, which we detected in persons born during 1940–2008 (Appendix Figure 7, panel A). Among the 35 persons born before 1980 and found to be positive for specific cellular immune responses, 28 (80%) had no detectable NAb (<1/4). However, 67.9% (19/28) persons showed IFN-γ responses in the ELISpot assay (Appendix Figure 7, panel B).

## Conclusions

We evaluated residual VTT immunity in the population of China across >5 birth decades. Our and other studies suggest that antibody responses against vaccinia virus after vaccination can be long-lived (*5*–*7*). We observed a low prevalence (28.7% [137/478]) of NAb against VTT in persons born before 1980, which is consistent with a previous study in the population of China (*8*). Our data demonstrate that 71.4% of the 35 tested participants born before 1980 had VTT-specific memory B-cell responses. Those memory B-cells can still rapidly differentiate into plasma cells and produce protective antibodies upon reinfection (*9*).

Smallpox vaccine–induced antibodies may protect against MPXV (*10*). Approximately 65.7% of the 35 participants born before 1980 that we tested had VTT-specific T-cell responses, which is consistent with previous reports that T-cell responses against vaccinia virus were maintained up to 51–75 years postimmunization and had a half-life of 8–15 years (*5*,*7*).

One limitation of our study is that it is a cross-sectional study. In addition, no information regarding smallpox vaccination or smallpox infection was available for the persons enrolled. Moreover, a small number of samples were tested for T- and B-memory cell responses.

In summary, we evaluated residual immune responses to VTT in the population of China and found that >65% of 35 tested persons born before 1980 showed memory B- and T-cell responses. However, the prevalence and NAb titers against VTT were low in this population. To protect the population from infection by MPXV and any other related pathogenic orthopoxviruses, safe and effective vaccines will be needed for all age groups.

Appendix Additional information about residual immunity from smallpox vaccination and possible protection from mpox, China.
