# Supplementary material for: Residual Immunity from Smallpox Vaccination and Possible Protection from Mpox, China
Source: Emerg Infect Dis. 2024 Feb;30(2):321–4. doi: 10.3201/eid3002.230542 (PMC10826747; doi:10.3201/eid3002.230542)
Supplement: Appendix — Additional information about residual immunity from smallpox vaccination and possible protection from mpox, China. [file 23-0542-Techapp-s1.pdf]

Article DOI: https://doi.org/10.3201/eid3002.230542

EID cannot ensure accessibility for supplementary materials supplied by authors. Readers who have difficulty accessing supplementary content should contact the authors for assistance.

Residual Immunity to Smallpox Vaccination and Possible Protection from Mpox, China

Appendix

Methods

Study design and participants

In this cross-sectional cohort study, we recruited 1070 healthy donors (583 males, 487 females) (Table 1), who came from Beijing city (n = 393), Shanxi province (n = 355), Heilongjiang province (n = 158), Hubei province (n = 101), or Shenzhen city (n = 63), China during regular health check-ups. 384 samples (243 males, 141 females) were collected in 2008, 576 samples (280 males, 296 females) were collected in 2014–2015, and 110 samples (60 males, 50 females) were collected in 2020–2021. Among these healthy donors, 478 (256 males, 222 females) were born in the period of 1930 to 1979, and 592 (327 males, 265 females) were born in the period of 1980 to 2008.

Venous blood was collected from participants and processed to isolate serum for immunological testing. Among these blood samples, 45 were used to isolate both plasma and peripheral blood mononuclear cell (PBMCs). All serum samples were inactivated at 56°C for 30 min before use.

Written informed consent was obtained from each individual. The study was approved by the Institutional Review Boards of Institute of Pathogen Biology, Chinese Academy of Medical Sciences (2013-IPB-03, IPB-2021–15).

Vaccinia virus Tiantan strain (VTT) Purification

Vero cells were infected with VTT at MOI of 0.05 and incubated at 37°C for 2 h. After washing, DMEM medium containing 2% FBS was added, cells were incubated at 37°C until cytopathic effect (CPE) was observed. After aspirating supernatant, cell lysis buffer (10 mM Tris-HCl pH 8.0) was added to the cells. Cells were subjected to freeze (−80°C)-thaw (37°C) for three times, and then sonicated and centrifuged at 4°C, 1500 g for 15 min to remove the cell debris. The clarified supernatant was processed with serial ultracentrifugations (44,500 g for 80 min with 36% sucrose cushion; 24,000 g for 40 min with step-gradient of 24%, 28%, 32%, 36%, and 40% sucrose; 31,500 g for 60 min). The purified virus was suspended with 10 mM Tris-HCl, pH 8.0 and titrated by plaque assay.

Construction and purification of vaccinia virus recombinants rTV-Gluc

Gaussia luciferase gene was inserted into the thymidine kinase (TK) locus in VTT genome under the transcriptional control of the early/late promoter p7.5 using a homologous recombination method in 293T cells. The recombinant, attenuated, replication-competent VTT carrying the Gaussia luciferase gene (rTV-Gluc) was propagated, plaque purified, and titrated by plaque assay in Vero cells.

Serum and PMBC isolation

Venous blood was collected from participants and processed within 12 h to isolate serum. Among these blood samples, 45 were used for isolating both plasma and peripheral blood mononuclear cells (PBMCs). Plasma was separated by centrifugation at 300×g for 10 minutes and stored at −80°C until testing. PBMCs were isolated from blood using Ficoll-Paque PLUS (GE Healthcare, Chicago, IL) according to the manufacturer’s instructions (https://www.fishersci.com/shop/products/ficoll-paque-plus-media-2/p-3753315). Isolated PBMCs were frozen in 90% heat-inactivated fetal bovine serum (FBS, Hyclone, Northbrook, IL) supplemented with 10% DMSO (Sigma-Aldrich, St. Louis, MO, USA), and stored in liquid nitrogen before analysis.

Enzyme-linked immunosorbent assay (ELISA)

VTT-specific IgG antibody titers were evaluated using the enzyme-linked immunosorbent assay (ELISA). Briefly, VTT infected Vero cells were lysed using RIPA buffer (Solarbio, Beijing, China) and were quantified using Pierce BCA Protein Assay Kit (Thermo Fisher Scientific, USA). Total 1 μg lytic proteins were used as coating antigens. Serum samples were diluted 1/100 in 0.2% bovine serum albumin (BSA) and incubated for 1 h at 37°C. After washing with PBST, horseradish peroxidase -coupled goat anti-human Fc specific polyclonal IgG (Sigma Aldrich, St Louis, MO, USA) antibodies were added to the plates at a dilution of 1/60,000 with 0.2% BSA. After 1 h incubation at 37°C, the plates were washed and developed with 100 μL TMB Two-Component Substrate solution in each well (Solarbio). Reaction was stopped by adding 50 μL of stop solution (Solarbio). Optical density at 450 nm (OD450) was determined with a multifunctional microplate reader SpectraMax M5 (Molecular Devices, Sunnyvale, CA, USA). The same amount of lytic Vero cell without VTT infection was used as a negative control for each serum sample. VTT-specific IgG responses were calculated by subtracting the background before further analysis. To determine the cutoff values for the ELISA, we determined the mean values and SD of negative serum from individuals who were born after 1990. The cutoff values were determined by calculating the OD450 of the negative serum plus 3-fold the SD values, which were 0.1 for VTT-specific IgG.

Microneutralization Assay

Neutralization was measured of serum samples by the reduction in Gaussia luciferase-based vaccinia neutralization assay. Briefly, a serial 2-fold dilutions of serum samples (starting at 1/4) was preincubated with an equal volume of rTV-GLuc virus (1000 plaque-forming units [pfu]) (Appendix Methods) for 1 h at 37°C. Then the virus-serum mixture was added to Vero cells (American Type Culture Collection number CCL-81) and incubated for 1 h. After washing, 200 μL of DMEM medium containing 2% FBS was added to Vero cells. After incubation for 24 h, 40 μL of culture supernatant was aspirated and mixed with 60 μL Coelenterazine-h substrate (Promega, Madison, WI, USA). The luminescence was measured using Steady-Glo Luciferase

Assay System (Promega). The neutralization titers (NT50) were defined as 50% luminescence reduction of the reciprocal of the highest dilution relative to the uninfected control cells.

Memory B cell ELISpot assays

Memory B cell ELISpot assays were performed using cryopreserved PBMCs with an ELISpot Flex: Human IgG (ALP) kit (Mabtech, NS, Sweden) following the manufacturer's instructions. Briefly, cryopreserved PBMCs were thawed and washed once with the RPMI-1640 medium containing 10% FBS and rested overnight before assay. PBMCs were pre-stimulated with mixture of R848 at 1μg/mL and rhIL-2 at 10 ng/mL for 48 h. After washing, total 2×10^5 PBMCs per well were added to the ELISpot plate pre-coated with 1×10^7 pfu heat-inactivated (56°C for 30 min) purified vaccinia virus. Lytic Vero cells were used as negative controls in each assay. Anti-human IgG mAb MT91/145 was used as positive control. Plates were incubated for 24 h at 37°C. After removing PBMCs, the plates were washed. Detection antibody diluted in 0.5% PBS-fetal calf serum was added and incubated for 1 h at room temperature. After washing, plate was added with streptavidin-ALP and incubated for 1 h at room temperature. Substrate solution were then added to the plate and developed until distinct spot emerges. The color development was stopped by washing in tap water. After the plate dried, the plate was inspected and counted spots in an AID ELISPOT reader system (AID Diagnostika GmbH, Strassberg, Germany). Mean spots of the negative control wells were subtracted from the test wells to quantify the intensity of VTT-specific memory B cell responses. The results were presented as Spot Forming Unit per 10^6 PBMC (s.f.u./10^6 PBMC). Memory B cell responses were considered positive if the mean spot count was ≥2-fold higher than the mean spot of the negative control and ≥10 s.f.u./10^6 PBMCs.

Ex-vivo Interferon-γ (IFN-γ) ELISpot assays

Memory T cell responses were evaluated using ex-vivo Interferon-γ (IFN-γ) ELISpot assays with a Human IFN-γ ELISpot kit (Mabtech) following the manufacturer's instructions. Briefly, cryopreserved PBMCs were thawed and rested overnight before assay. Plates pre-coated with human IFN-γ antibodies were washed with phosphate-buffered saline (PBS), and blocked

with RPMI1640 medium containing 10% FBS for 30 min at room temperature. Total 1×10^5 PBMC per well were stimulated in duplicates with 10^5 pfu inactivated (56°C for 30 min) purified VTT for 24 h at 37°C. Lytic Vero cells were used as negative controls in each sample. Phorbol myristate acetate (PMA)/ionomycin (Multi Science, Hangzhou, China) and known T cell epitope pools of human influenza (Flu) and Epstein-Barr virus (EBV) were used as positive controls. Spots were counted using an AID ELISPOT Reader System. Mean spots of the negative control wells were subtracted from the test wells to quantify the intensity of VTT-specific T cell responses, and the results were presented as Spot Forming Unit per 10^6 PBMC (s.f.u./10^6 PBMC). Memory T cell responses were considered positive if the mean spot count was ≥2-fold higher than the mean spot of the negative control and ≥20 s.f.u./10^6 PBMCs.

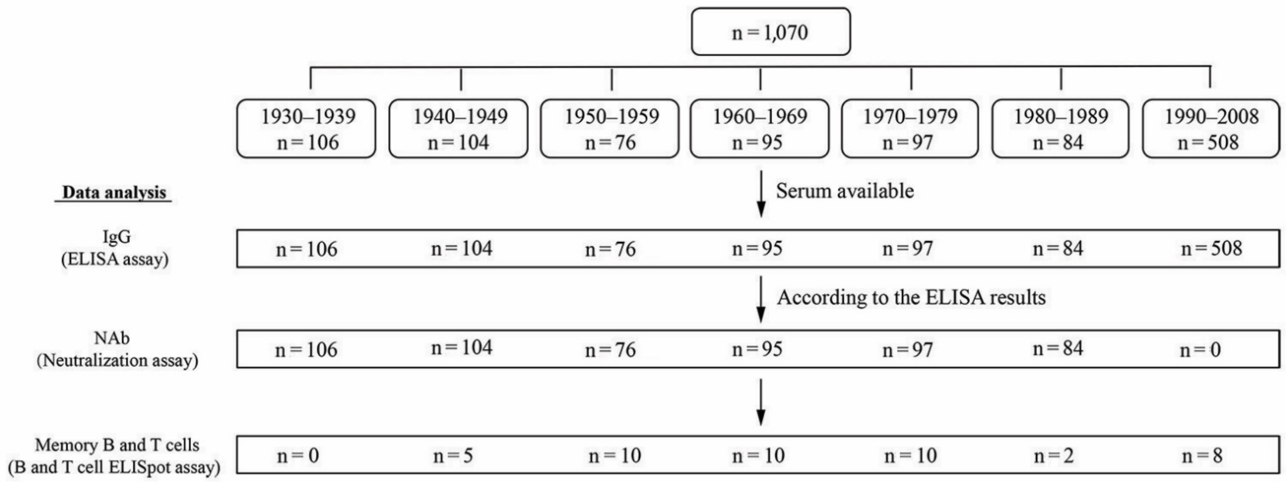

Appendix Figure 1. Flow diagram of the study.

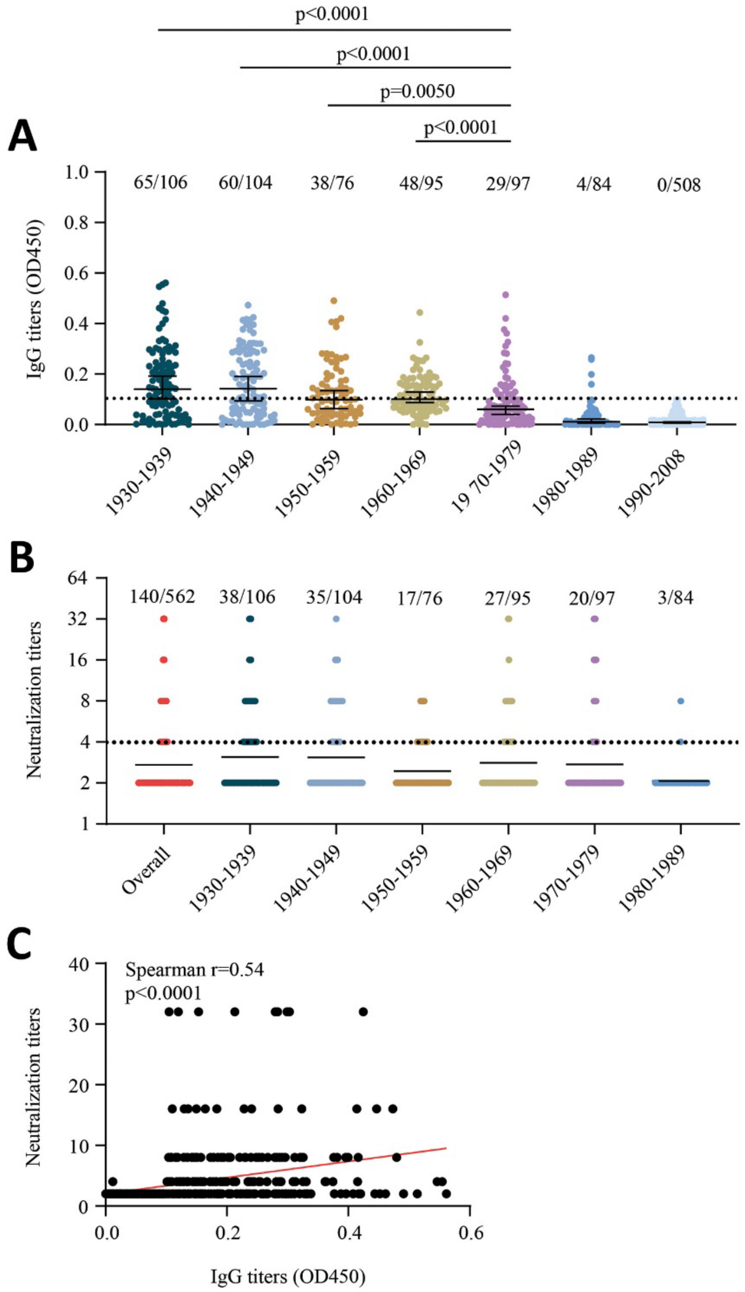

Appendix Figure 2. Serum IgG and neutralizing antibody responses against vaccinia virus Tiantan strain. (A) IgG titers against VTT according to the year of birth. (B) Neutralizing antibody titers against VTT according to the year of birth. (C) Correlation between neutralizing antibody and IgG titers against VTT in individuals born before 1990 (n = 562). IgG titers comparisons were performed using the Kruskal-Wallis one-way ANOVA test in different age groups. Spearman correlation analysis was performed.

VTT = vaccinia virus Tiantan strain, OD = optical density.

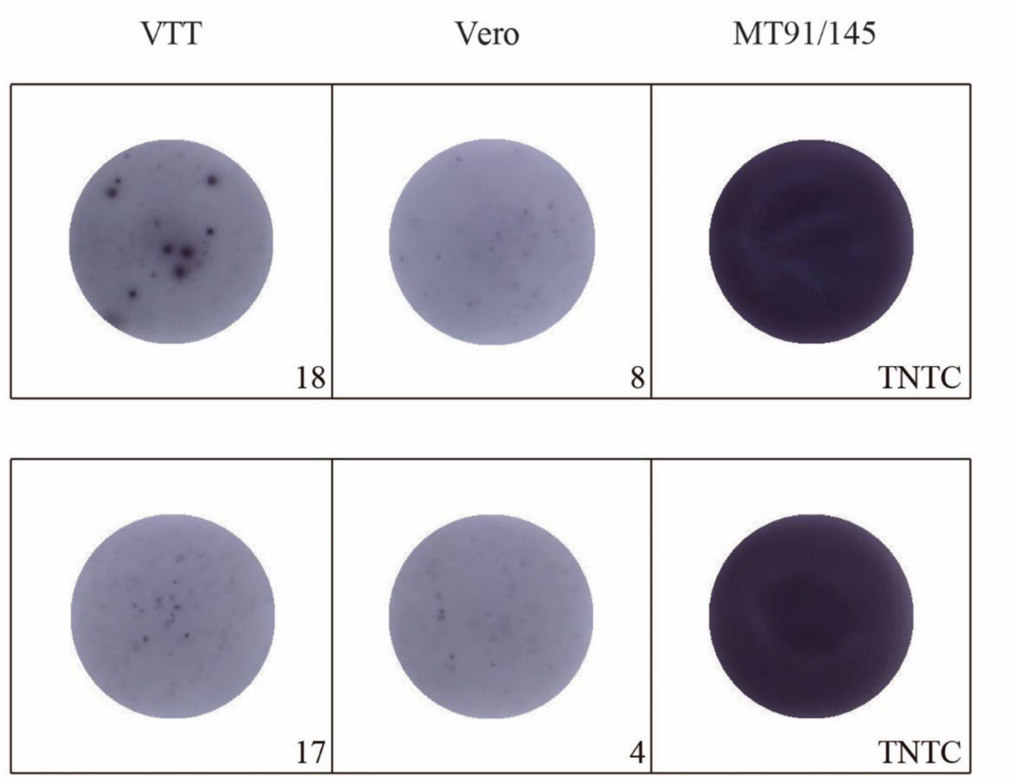

Appendix Figure 3. VTT-specific memory B cell responses. Two representative sample of the memory B cell ELISpot responses. Vero cell was used as the negative control. Anti-human IgG mAb MT91/145 was included as the positive control. TNTC = too numerous to count.

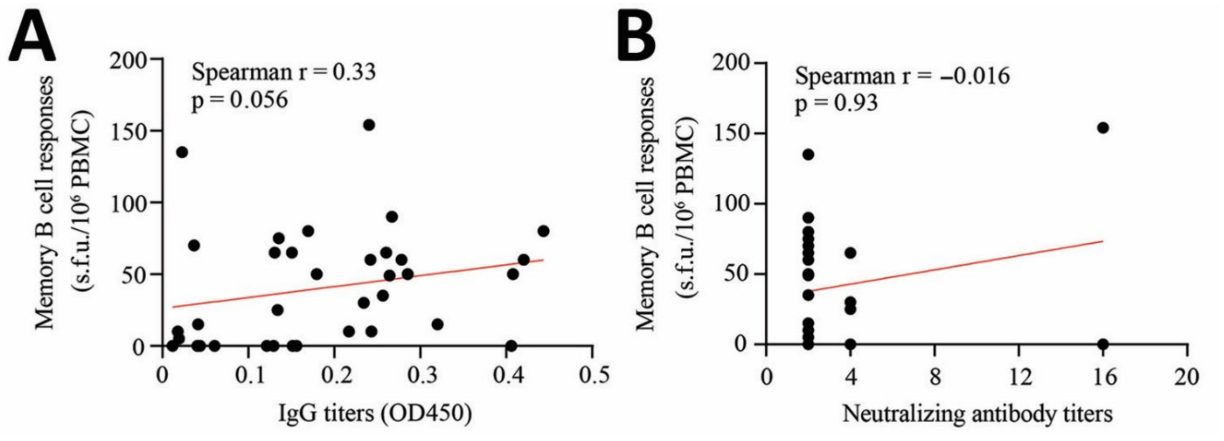

Appendix Figure 4. Correlation between memory B cell responses and antibody titers. Correlation between the magnitudes of memory B cell responses and IgG (A), and neutralizing antibody (B) titers against VTT (n = 35). Spearman correlation analysis was performed.

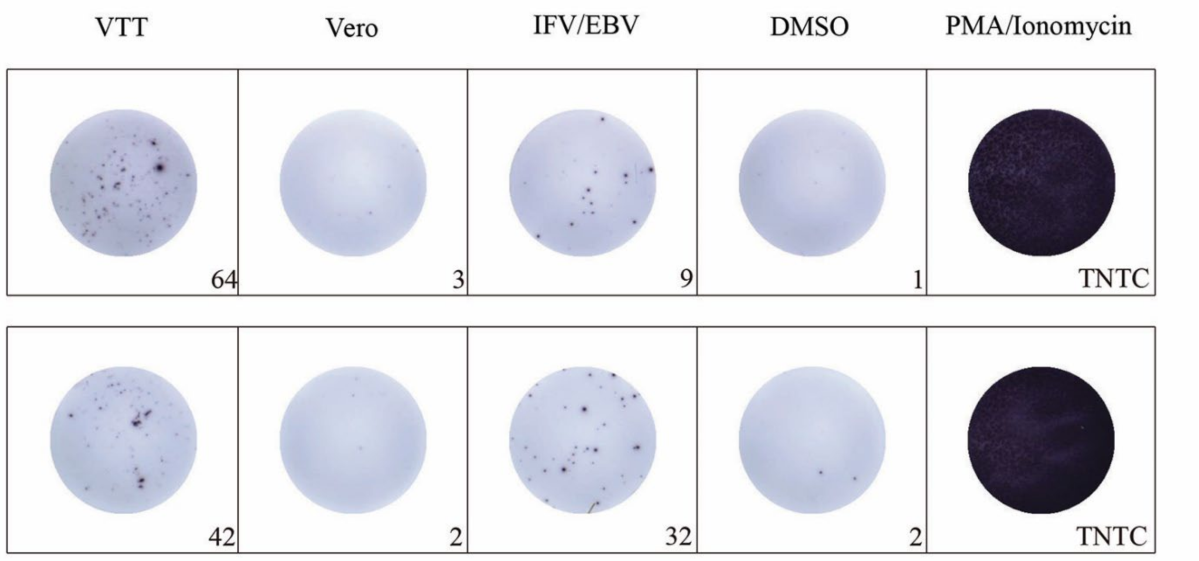

Appendix Figure 5. Two representative samples of the IFNγ ELISpot responses. Uninfected Vero cell serves as the negative control of vaccinia virus infection. DMSO serves as the negative control of IFV/EBV peptide pool. PMA/Ionomycin was assayed as the positive control.

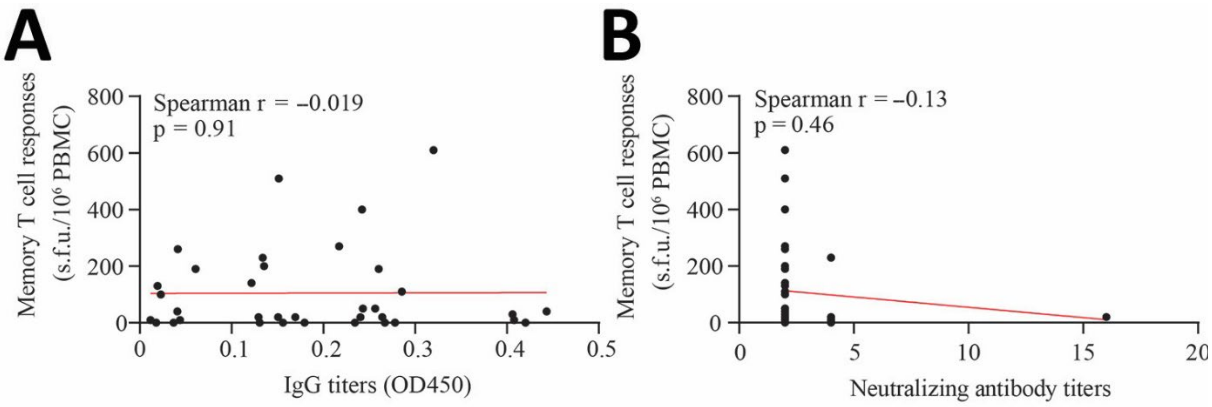

Appendix Figure 6. Vaccinia virus-specific memory T cell responses. (A) Magnitude of IFNγ T-cell responses against IFV/EBV for each individual (n = 45). (B) Magnitude of IFN-γ T cell responses in ELISpot for 35 individuals who had been evaluated of neutralizing antibody levels and were born before 1980. The dotted lines indicate the detection limit of the assays. IFV = Influenza virus, EBV = Epstein-Barr virus, TNTC = too numerous to count.

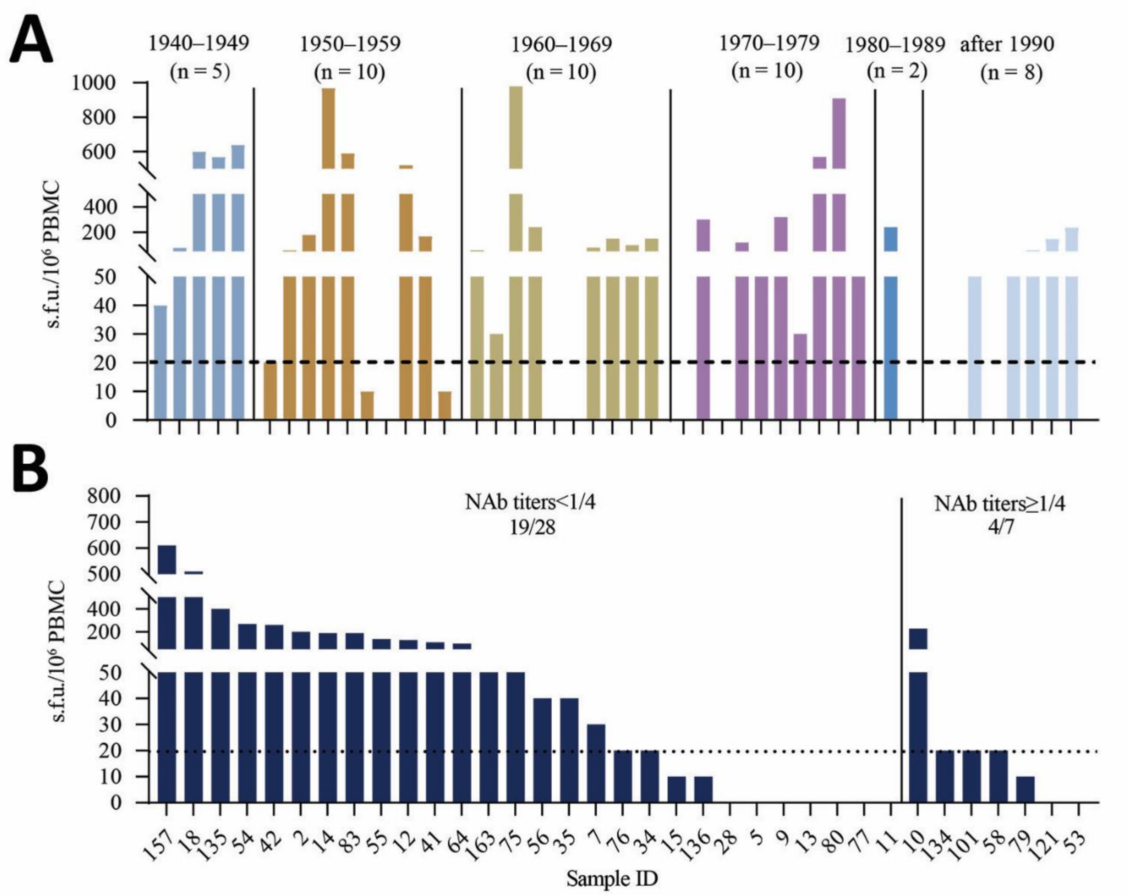

Appendix Figure 7. Correlation between memory T cell responses and IgG titers. Correlation between the magnitude of IFNγ ELISpot responses and IgG (A) and the neutralizing antibody (B) titers against VTT (n = 35). Spearman correlation analysis was performed.
